# Estimation of Dynamic Canopy Variables Using Hyperspectral Derived Vegetation Indices Under Varying N Rates at Diverse Phenological Stages of Rice

**DOI:** 10.3389/fpls.2018.01883

**Published:** 2019-01-15

**Authors:** Mairaj Din, Jin Ming, Sadeed Hussain, Syed Tahir Ata-Ul-Karim, Muhammad Rashid, Muhammad Naveed Tahir, Shizhi Hua, Shanqin Wang

**Affiliations:** ^1^College of Resources and Environment, Huazhong Agricultural University, Wuhan, China; ^2^Key Laboratory of Arable Land Conservation (Middle and Lower Reaches of Yangtze River), Ministry of Agriculture, Wuhan, China; ^3^Department of Agronomy, University of Agriculture Faisalabad, Burewala, Pakistan; ^4^Key Laboratory of Soil Environment and Pollution Remediation, Institute of Soil Science, Chinese Academy of Sciences, Nanjing, China; ^5^Plant Breeding and Genetics, Nuclear Institute for Agriculture and Biology, Faisalabad, Pakistan; ^6^Department of Agronomy, PMAS-Arid Agriculture University Rawalpindi, Rawalpindi, Pakistan

**Keywords:** dynamic canopy variables, hyperspectral reflectance, rice, phenology, N-nutrition

## Abstract

Non-destructive and rapid estimation of canopy variables is imperative for predicting crop growth and managing nitrogen (N) application. Hyperspectral remote sensing can be used for timely and accurate estimation of canopy physical and chemical properties; however, discrepancies associated with soil and water backgrounds complicate the estimation of crop N status using canopy spectral reflectance (CSR). This study established the quantitative relationships between dynamic canopy nitrogen (CN) status indicators, leaf dry weight (LDW), leaf N concentration (LNC), leaf N accumulation (LNA), and CSR-derived new hyperspectral vegetation indices (HVIs), and to access the plausibility of using these relationships to make in-season estimations of CN variables at the elongation (EL), booting (BT), and heading (HD) stages of rice crop growth. Two-year multi-N rate field experiments were conducted in 2015 and 2016 in Hubei Province, China, using the rice cultivar Japonica. The results showed that the sensitive spectral regions were negatively correlated with CN variables in the visible (400–720 nm and 560–710 nm) regions, and positively correlated (*r* > 0.50, *r* > 0.60) with red and NIR (720–900 nm) regions. These sensitive regions are used to formulate the new (SR_777/759_, SR_768/750_) HVIs to predict CN variables at the EL, BT, and HD stages. The newly developed stepwise multiple linear regression (SMLR) models could efficiently estimate the dynamic LDW at the BT stage and LNC and LNA at the HD stage. The SMLR models performed accurately and robustly when used with a validation data set. The projected results offer a suitable approach for rapid and accurate estimation of canopy N-indices for the precise management of N application during the rice growth period.

## Introduction

Nitrogen (N) is an essential nutrient for crop growth and productivity. It is an indispensable constituent of chlorophyll, as well as proteins associated with leaf color, plant vigor, plant N status, crop yield, and quality. Nitrogen insufficiency in rice leads to major issues such as small leaf size, lower chlorophyll and protein content, lower dry matter accumulation, and leaf expansion (Ata-Ul-Karim et al., 2013, 2014, 2016, 2017a; Wang et al., 2014). However, an excessive N supply leads to lower N use efficiency, creating health and environmental hazards (Liu et al., 2013; Bodirsky et al., 2014). In-season efficient N management, both in term of rates and stage of application, is vital for rice crop production and environmental sustainability (Tahir Ata-Ul-Karim et al., 2016). Therefore, precise N management and accurate estimation of crop N status has been the most prominent issue in modern crop production; not only for economic reasons, but also to curtail the environmental hazards associated with over-applying N fertilizer (Zhao et al., 2016).

The traditional destructive methods for measuring biophysical and biochemical parameters of crop are laborious and time consuming. CMR have become a standard for real time non-destructive diagnosis of crop N status. However, it is not practical to apply this method across large fields (Zhao et al., 2018). Additionally, the CMR are actually based on absorption by chlorophyll instead of N. Several factors, such as leaf thickness, leaf position on the plant, leaf specific weight, measurement location on a leaf, plant growth, cultivar (Muñoz-Huerta et al., 2013), as well as environmental stress and solar radiation (Ata-Ul-Karim et al., 2016; Yuan et al., 2016) can affect the CMR considerably. The success of precision N management requires the development of rapid, non-destructive technology to monitor and estimate crop N status throughout the growing season (Li et al., 2018).

Remote sensing has also been widely used as a rapid and non-destructive tool to monitor biophysical and biochemical parameters and to estimate in-season crop N status. Optical remote sensing has a long history with N estimation. However, estimation of biochemical parameters is difficult at the resolution of individual leaves; therefore, estimation of integrated biochemical and biophysical properties at the canopy level are preferred (Jones et al., 2007). Crop canopy sensors have been used effectively in precision agriculture for estimation of crop growth, chlorophyll content, and crop N status (Samborski et al., 2009; Diacono et al., 2013). Active canopy sensors that have their own light sources are weather independent. Moreover, hyperspectral canopy sensors provide effective information for the estimation of N status in field crops (Freek et al., 2012). Hyperspectral reflectance assessment of the crop canopy offers direct and instant information for in-season N application (Xavier et al., 2006). Extraction and identification of sensitive bands from hyperspectral reflectance, that particularly contains information about plant N distribution has the potential for construction of HVI (Stroppiana et al., 2009). Active canopy sensor provides the facility to select suitable bands (red, red edge, and NIR region) for construction of HVIs having great potential for estimation of rice biomass and nitrogen accumulation across different growth stages.

As a key plant organ, the leaf plays a vital role in photosynthesis; thus, leaf N status, rather than that of the whole canopy, is more reliable and suitable for N management (Jongschaap and Booij, 2004; Mistele and Schmidhalter, 2008). The association between the plant canopy and solar radiation give the spectral response, providing a basis for construction of several vegetation indices for the successful estimation of PNC. Unfortunately, factors like soil background, atmospheric resistance, and vegetation canopy structure complicate studies of plant CSR.

Most vegetation indices perform poorly due to saturation and loss of sensitivity of above ground biomass, especially for closed canopies (Mokhele and Ahmed, 2010). HVIs using NIR and red bands have been widely used to resolve canopy saturation issues. The hyperspectral indices account for reflectance information from all possible combinations rather than relying on specific bands. Indices based on the green, (500–570 nm) red, far red (650–680 nm), red edge (700–720 nm), and NIR bands have been used to predict the chlorophyll content, N status, N rate, LNC, and PNC at the leaf level (Xue et al., 2004; Zhao et al., 2005a). Vegetation indices based on 530–560, 630–660, and 760–900 nm bands have been strongly correlated to N status in rice (Serrano et al., 2000). Kooistra et al. (2005) showed that the hyperspectral reflectance in the red-edge, visible, and NIR regions might provide additional information about chlorophyll and N estimation. Extraction and identification of sensitive bands from hyperspectral reflectance, particularly those that contain information about canopy variables, is the basis for construction of reliable vegetation indices (Stroppiana et al., 2009). Simple statistical analysis always suffers from the problem of over-fitting due to the number of bands (Xu et al., 2013). To overcome the problems of over-fitting and co-linearity, and to improve model accuracy, a SMLR analysis is commonly applied. Li et al. (2014) found that the stepwise linear regression analysis successfully extracts the important bands related to plant N-status. In order to deal with the complicated phenomenon of high dimensionality and redundancy in the processing of hyperspectral data, it is necessary to extract the sensitive bands for in-season estimation of LDW, LNC, and LNA in rice. More specifically, few studies have been carried out to analytically investigate the published indices for CN status indicators (LDW, LNC, and LNA) collectively at the critical EL, BT, and HD stages of rice growth, rice being a crop that is subject to significant soil and standing water backgrounds. The standing water background expand the spectral reflectance range for rice.

The objectives of the present study were to identify the specific spectral bands for CN status indicators (LNA, LNC, and LDW), to develop new HVIS based on newly identified sensitive spectral regions for estimation of CN variables, and to monitor dynamic CN status indicators across phenological stages of rice growth using static and dynamic models based on HVIS under different N-rates.

## Materials and Methods

### Experiment Site, Design, and Crop Management

Field experiments were conducted at the experimental station of Huazhong Agricultural University in Wuxue, China (30°060N, 115°350E) in 2015 and 2016. The region has an annual average temperature of 17.7°C and annual average precipitation of 1903 mm, with a subtropical moist monsoon climate. Summer is the driest season and autumn the wettest for the area around the experimental station.

Two multi-N rate (0–293 kg N ha^-1^ as Urea) field experiments were conducted using Japonica rice cultivar Shenliangyou 5814 (Table 1). Treatments were laid out in a randomized complete block design (RCBD), with three replicates. The fertilizers, SSP (P_2_O_5_ content 12%) @ 90 kg ha^-1^ and potassium (K_2_O content 60%) @ 180 kg ha^-1^, were applied before transplantation. The area of each plot was 20 m^2^. A 40 cm wide ridge covered with plastic film was inserted to a soil depth of up to 30 cm to separate the plots and avoid any exchange of water or fertilizer. Thirty-day-old seedlings were planted with spacing of 0.24 m × 0.30 m to maintain a planting density of approximately 26 hills per m^2^. Weeds, insects, and diseases were strictly monitored throughout the growing period. No major attacks of weeds, disease, pests, or inclement of weather were recorded during the growing season. Weeds were removed manually and due to low insect pressure no pesticide was required.

**Table 1:** Basic information about the two field experiments conducted during 2015 and 2016.

Experiment-1 (2015)			
N (kg ha^-1^)	N distribution (%) and stages	Sensing and sampling stage	Sensing and sampling dates
N0 (0)	PP (20%)	AT	11-July
N1 (45)	AT (25%)	TL	20-July
N2 (83)	BT (55%)	EL	31-July
N3 (128)		BT	15-August
N4 (165)		HD	30-August
N5 (210)		MT	15-September
N6 (248)			
N7 (293)			
**Experiment-2 (2016)**			
N0 (0)	PP (20%)	AT	10-July
N1 (45)	AT (25%)	TL	19-July
N2 (83)	BT (55%)	EL	28-July
N3 (128)		BT	11-August
N4 (165)		HD	28-August
N5 (210)		MT	11-September
N6 (248)			
N7 (293)			

*Nitrogen (N) fertilizer were applied 20, 25, and 55% at PP, AT, and BT, respectively, as Pre-planting (PP), After Transplanting (AT), Tillering (TL), Elongation (El), Booting (BT), Heading (HD), Maturity (MT).*

### Measurements and Sampling

#### Canopy Spectral Reflectance Data Collection

Spectral reflectance measurements were acquired using a portable FRTM, an analytical spectral device (ASD, Boulder, CO, United States) that covers the 350–2500 nm spectral range, between 10:00 and 14:00 h China Standard Time (UTC+8) under clear and cloudless skies (Table 1). Three spectral measurements, two at plot corners and one at plot centers, in both 2015 and 2016, were recorded with the spectro-radiometer sensor head one meter above the rice hills, with a nadir of 25°. Spectral calibration was carried out using radiance from a Spectralon reference panel (BaSO_4_), and was conducted every 30 min. For each plot, thirty spectra were exported and averaged using RS2 (ASD, Boulder, CO, United States) software.

#### Pre-processing of Hyperspectral Reflectance

Noise and background water absorption affect the absorption of canopy spectra; therefore, the raw spectra were reduced in different sections (1341–1439, 1791–1959, and 2401–2500 nm) (Abdel-Rahman et al., 2010). In the current study, we focused on the spectral regions (350–1000 nm) that contained key information on rice growth. Every fifth waveband was averaged into one spectral band variable to reduce the hyperspectral dimension over all the hyperspectral bands.

#### Leaf Nitrogen Indicators Measurements

For analysis of N indicators, the above ground parts of the rice plants were sampled after every spectral reflectance measurements at Tillering (TL), EL, BT, HD, and maturity (MT) stages. Samples of five hills per plot were collected in both 2015 and 2016. After washing, roots were clipped and the samples were divided into their plant organs, leaves, stems, and heads. The samples were dried at 105°C for half an hour and then at 70°C in a WGL forced air drying oven (WGL-125B), until a constant weight was achieved. Once dry, all of the samples from tilling to maturity stages were weighed for LDW and LNC analysis. LNC were determined using a Flow Injection Analyzer (AA3HR, AutoAnalyzer, SEAL, Norderstedt, Germany) system and LNA were calculated as the product of N concentration per unit dry weight in tissue and dry weight per unit ground area by the following formula:

(1) LNA = LNC × LDW

### Hyperspectral Vegetation Indices

Raw reflectance data were used to calculate the HVIs listed in Table 2.

**Table 2:** Descriptions and formulas of vegetation indices investigated for leaf nitrogen status indictor (LDW, LNC, and LNA) during 2015 and 2016.

Indices	Formulas	Reference
SR768,750	R768/R750	This Study
SR777,750	R777/R550	This Study
SR810,560	R810/R560	Xue et al., 2004
SR777,759	R777/R759	Xue et al., 2004
SR810,660	R810/R660	Chu et al., 2014
SR750,705	R750/R705	Gitelson and Merzlyak, 1994b
ND860,560	R860–R560	Chu et al., 2014
ND860,720	R860–R720	Fava et al., 2009
ND759,732	R759–R732	Hansen and Schjoerring, 2003
DD	(R750 – R720) – (R700 – R670)	Le Maire et al., 2004
NDI780	(R780 – R710)/(R780 – R680)	Datt, 1998
NDI850	(R850 – R710)/(R850 – R680)	Datt, 1998
NDVI800	(R800 – R700)/(R800 + R700)	Gitelson and Merzlyak, 1994a
NDVI780	(R780 – R550)/(R780 + R550)	Gitelson et al., 1996
NDVI760	(R760 – R708)/(R760 + R708)	Steddom et al., 2003
ND_705_	(R750 – R705)/(R750 +R705)	Sims and Gamon, 2002
MTCI	(R750 – R710)/(R710 – R680)	Dash and Curran, 2004
MCARI	[(R750 – R705) – 0.2^∗^(R750 –R550)](R750/R705)	Wu et al., 2008
MSR	(R750/R705 – 1)/SQRT(R750/R705 + 1)	Wu et al., 2008
DD/MSAVI	DD/MSAVI	Haboudane et al., 2008

*Simple Ratio (SR), Normalized difference (ND), Double difference index (DD), Normalized difference index (NDI), Normalized difference vegetation index (NDVI), Normalized difference vegetation index *705* (ND_*705*_), The MERIS terrestrial chlorophyll index (MTCI), Modified chlorophyll absorption in reflectance index (MCARI), Modified simple ratio (MSR), Modified soil adjusted vegetation index (MSAVI), DD/MSAVI.*

### Statistical Analysis

The data on CN status indicators at different growth stages from each sensing and sampling date were subjected to analysis of variance (ANOVA) using the GLM procedure in IBM SPSS Version19.0 (IBM Corporation, Armonk, NY, United States). The means were compared for difference in treatments using a least significant difference (LSD) test at the 5% level of significance. The coefficient of correlation (*r*) for the relationships between canopy spectral region (400–900 nm) and CN status indicators at each growth stage were calculated to identify the sensitive spectral ranges using Microsoft Excel 2010 (Microsoft Corporation, Redmond, WA, United States). The univariate non-linear regression (Power) between HVIs and CN status indicators were developed using Origin 8.0 (Originlab Corporation, United States). Coefficients of determination (*R*^2^) from regression analyses were used to identify HVIs for predicting CN status indicators at each growth stage.

### Development and Validation of Dynamic Models

A correlation and univariate non-linear regression might be insufficient due to interrelation of dynamic CN status indicators over phenology. SMLR were used to identify and quantify the relationships of HVIs and CN status indicators. Observations were made of the experimental plots (*n* = 48) in both 2015 and 2016. The two year data set was divided into calibration data (70%), used in the SMLR model for estimation, and validation data (the remaining 30%). Forward selection and backward elimination were combined in the SMLR method. The HVIs and CN status indicators were input in SMLR to predict SMLR models for each phenological stage, using the calibration data set. The best phenological stage for predicting CN status indicators throughout the growth period, and corresponding most accurate SMLR model, were calculated using MATLAB and Statistics Toolbox Released 2009b, (The Mathworks, Inc., Natick, MA, United States). Vasques et al. (2009) reported that the SMLR method had the potential for effective selection and analysis of hyperspectral reflectance data.

The validation was based on the root mean squared error (RMSE) and coefficient of determination (*R*^2^). The *RMSE* constitutes how good the regression models (best-fit function) are at capturing the relationship between biophysical and biochemical parameters and HVIS (Yao et al., 2010). Higher *R^2^* and lower *RMSE* will indicate that the model works with precision and accuracy for the prediction of CN status indicators.

(2) $$\text{RMSE}=\sqrt{\frac{1}{n}\times \sum_{i=1}^{n}{\left(y_{i}-{\hat{y}}_{i}\right)}^{2}}$$

where, *y_i_* and *y_i_* are the measured and predicted, values of the variables, and, *n* is the number of samples (Feng et al., 2014).

## Results

### Variations in Canopy Nitrogen Status Indicators (LDW, LNC, and LNA) Over Phenology of Rice Under Varied N Rates

**FIGURE 1:**
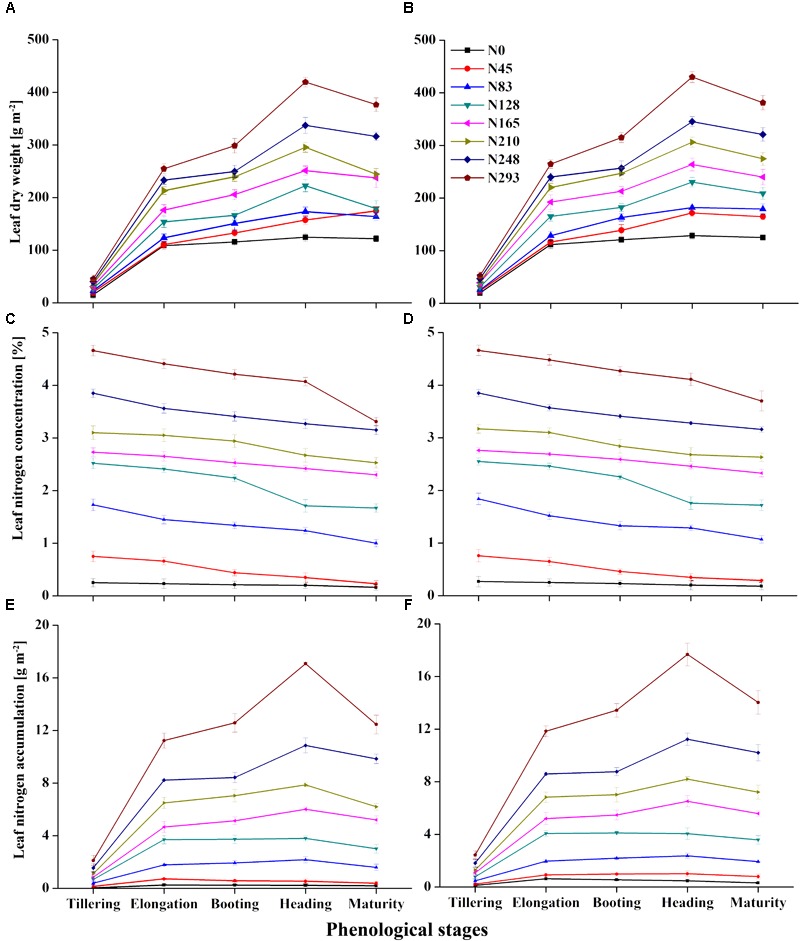
Variation in leaf nitrogen status indicator leaf dry weight (LDW) **(A,B)**, leaf nitrogen concentration **(C,D)** and leaf nitrogen accumulation **(E,F)** in 2015 and 2016, respectively, over phenology of rice.

The leaf N status indicators were more variable during the EL, BT, and HD stages, than during the TL and MT stages. As shown in Figures 1A,B,E,F, a higher N rate resulted in higher LDW and LNA. The stages LDW and LNA in particular varied substantially in response to varying N rates. In each growing season, minimum variations in the ranges of LDW and LNA were observed during the vegetative stage, with maximum variation observed during the reproductive stage. LNC varied markedly across the growth stages (Figures 1C,D), and was minimal at the early stages of crop growth before tiller initiation in both 2015 and 2016. The LNC was negatively affected after the initiation of tiller, especially from EL to MT, showing a significant decrease during this period. LDW, LNC, and LNA under varied N rates, at TL and HD, respectively, showed values of 29.86–247.70 g m^-2^ (Figure 1A), 2.45–1.99% (Figure 1C), and 0.86–6.07 g m^-2^ for 2015 (Figure 1E). The same variables showed values of 34.79–257.22 g m^-2^ (Figure 1B), 2.48–2.02 % (Figure 1D), and 1.03–6.46 g m^-2^ for 2016 (Figure 1F). LDW and LNA improved from tillering, elongation, and heading. For particular growth stages, LDW and LNA increases were pronounced at booting and heading, respectively. LDW and LNA values increased from 194.55 to 247.70 g m^-2^, and 4.96 to 6.07 g m^-2^, respectively, in 2015, and from 204.58 to 257.22 g m^-2^, and 5.32 to 6.45 g m^-2^, respectively, in 2016, but declined after that, during the grain filling stages. LNC decreased from BT and HD in both 2015 and 2106, with average LNC values falling from 2.16 to 1.99 and from 2.17 to 2.02, respectively.

### Changes in CSR Under Varied N Rates and Growth Stages

**FIGURE 2:**
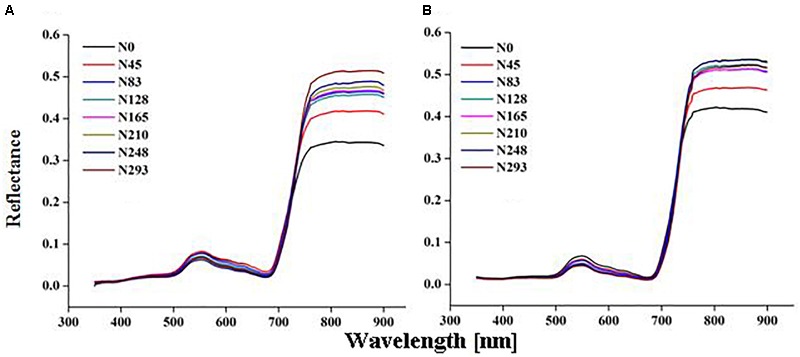
Change in canopy reflectance spectra under different nitrogen (N) fertilization rates of rice in 2015 **(A)** 2016 **(B)**.

**FIGURE 3:**
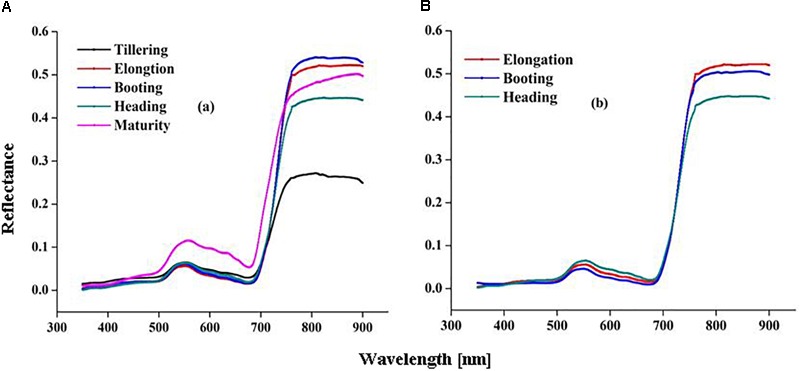
Variation in canopy spectral reflectance (CSR) over growth stages of rice in 2015 **(A)** and 2016 **(B)**.

Marked variation in CSR was recorded under different N rates in present study (Figures 2A,B). The significant changes in CSR were recorded at the EL, BT, and HD stages in both 2015 and 2016. The behavior of different spectrum regions changed in a manner analogous to variations observed for other green plants under varied N rates. Reflectance increased in the NIR region (>720 nm), while it decreased in the visible (400–720 nm) and ultraviolet (350–400 nm) regions of the spectrum as N varied from 0 to 293 kg N/ha during crop development. The variations in reflectance in the NIR region (780–900 nm) under N rates over different growth stages were quite obvious in both 2015 and 2016 (Figures 3A,B). At critical growth stages the CSR was not constant, but consistently changed as N rates varied. The decreasing trend in the visible region and the increasing trend in the NIR region of CSR, were apparent in 2016 from the elongation to the heading stages (Figure 3B), with the exception of the BT stage in 2015, which had more reflectance in NIR region than the EL and HD stages (Figure 3A). The decline of CSR in the visible region at early (TL) and late (MT) stages in 2015 (Figure 3A) was the reason that these two stages were excluded from 2016 (Figure 3B) CSR observation. Similar patterns of decreased CSR in the visible region and increased CSR in the NIR region were observed at the EL, BT, and HD stages in both years (Figures 3A,B). The crop phenological stages, up to senescence, can be clearly identified based on the behavior of the red and green parts of the visible spectrum. In this study CSR exhibited prominent negative variation in the visible (530–560 nm), and red edge (700–780 nm) region. However, positive variation in the NIR region (680–900 nm) specifically at 780, 800, and 850 nm wavebands, under different N application provides the basis for the construction of HVIs for different N applications.

### Relationships Between CSR and Canopy Nitrogen Status Indicators (LDW, LNC, and LNA) During Rice Growth Under Varied N Rates

**FIGURE 4:**
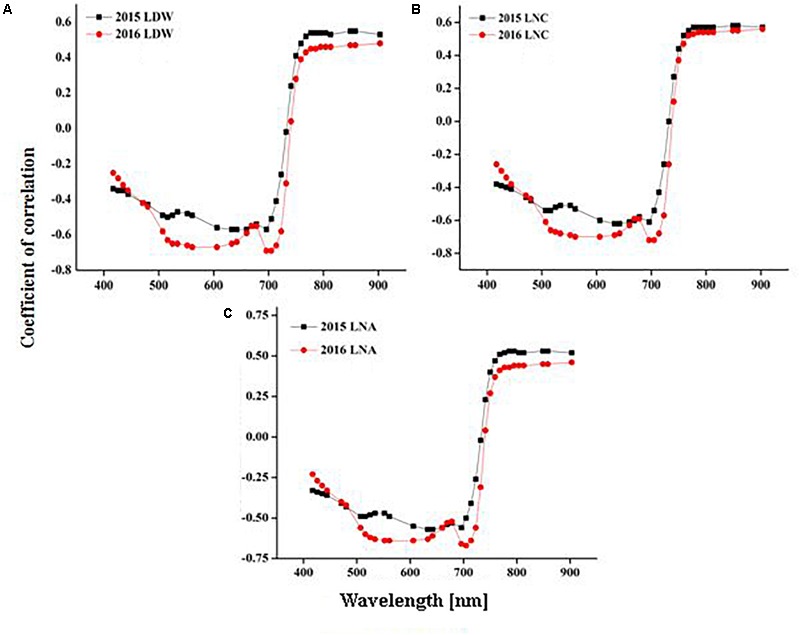
Coefficient of correlation between leaf nitrogen status indicators [LDW, **(A)**, LNC, **(B)** and LNA, **(C)**] and CSR over rice growth in 2015 and 2016.

The CSR responded to the wide variation in CN status indicators (LDW, LNC, and LNA) across the various stages of rice growth. The correlation coefficient (*r*) between all the CSR bands and CN status indicators were plotted over rice growth periods under varied N rates (Figures 4A–C).

The results showed that CSR exhibited negative correlations (*r* < –0.30 to < –0.40) with LDW, LNC, and LNA in the visible (400–720 nm) and (560–710 nm) regions, while positive correlations (*r* > 0.50 to *r* > 0.60) were expressed in the red and NIR region (720–900 nm). In the visible spectrum, blue (400–480 nm), green (500–560 nm), red (600–730 nm), and red edge (740–760 nm) regions were negatively correlated (*r* < –0.30 to –0.70) while NIR (760–780 nm) and (780–900 nm) regions were positively (*r* > 0.50) correlated to LDW over the growing period (Figure 4A). LNC in the blue (400–480 nm), green (500–560 nm), and red (600–730 nm) parts of visible spectrum were negatively (*r* < –0.5 to < –0.65) correlated, while the red edge (740–760 nm) and NIR (760–900 nm) regions were positively correlated (*r* < 0.50 to 0.55) to CSR over the growth period in both 2015 and 2016 (Figure 4B). For LNA in the visible region the blue bands (400–480 nm) exhibited the lowest correlation (*r* < –0.40) with CSR. The green (500–560 nm) and red (600–730 nm) regions of CSR had correlation values of *r* < –0.50 and *r* < –0.55, respectively. The coefficient of correlation was positively (*r* < 0.50) related for the red edge (740–760 nm), and significantly associated (*r* > 0.50–0.60) for the NIR (760–900 nm) region of CSR for LNA in both 2015 and 2106 (Figure 4C).

### Relationship Between HVIs and Canopy Nitrogen Status Indicators (LDW, LNC, and LNA) Over Growth Stages Under Varied N Rates

Comprehensive correlation analyses were conducted at three phenological stages to evaluate the performance of twenty HVIs with CN status (LDW, LNC, and LNA indicators (Table 3). Marked variation in LDW over growth from TL to MT resulted in a similar pattern of fluctuation in coefficient of correlation (*r*) between HVIs and LDW. The performance of HVIs varied with CN status indicators. Top ten HVIs showed the highest coefficient of correlation (*r*) for LDW among the twenty indices used in this study. Overall, at three critical stages (EL, BT, and HD) the four HVIs, MTCI, MSR, NDVI_800_, and SR_810/560_ had better correlations for LDW. Generally, a gradual decrease in coefficient of correlation at the three stages was noted, but some HVIs expressed inconsistent behavior for LDW over phenology. For LNC among the twenty HVIs, eight HVIs had a superior relationship with LNC at early (EL), rather than BT and HD stages. The decreasing trend in coefficient of correlation was observed over three critical stages (EL, BT, and HD) for LNC over two years. For LNA, among eleven HVIs, the top five HVIs, SR_768/750_, SR_777/750_, SR_810-560_, ND_860-720_, and MTCI performed quite well (*r* > 0.80 – >0.60) at three critical stages (EL to HD) for LNA.

**Table 3:** Correlation coefficient (*r*) between leaf N status indicators (LDW, LNC, and LNA) over growth stages of rice in 2015 and 2016.

	Coefficient of correlation (*r*)									
		LDW			LNC			LNA		
HVls	Year	EL	BT	HD	EL	BT	HD	EL	BT	HD
SR768/750	2015	–	–	–	0.91	0.91	0.87	0.85	0.79	0.77
	2016	–	–	–	0.90	0.87	0.80	0.84	0.81	0.75
SR 777/750	2015	–	–	–	–	–	–	0.84	0.79	0.77
	2016	–	–	–	–	–	–	0.84	0.81	0.75
SR777/759	2015	0.74	0.61	0.58	–	–	–	–	–	–
	2016	0.79	0.64	0.46	–	–	–	–	–	–
SR810/560	2015	0.73	0.50	0.54	0.91	0.80	0.82	0.85	0.74	0.70
	2016	0.81	0.73	0.62	0.90	0.82	0.72	0.83	0.64	0.71
SR810/660	2015	0.66	0.41	0.51	0.89	0.75	0.80	0.84	0.56	0.55
	2016	0.78	0.54	0.49	0.92	0.64	0.58	0.79	0.56	0.70
ND860-560	2015	0.70	0.46	0.54	–	–	–	0.82	0.74	0.70
	2016	0.81	0.73	0.62	–	–	–	0.82	0.61	0.71
ND860-720	2015	–	–	–	–	–	–	0.75	0.75	0.55
	2016	–	–	–	–	–	–	0.65	0.77	0.71
ND759-732	–	–	–	–	0.76	0.84	0.79	–	–	–
	–	–	–	–	0.86	0.83	0.65	–	–	–
NDI850	–	0.70	0.61	0.56	0.88	0.82	0.84	–	–	–
	–	0.76	0.71	0.67	0.90	0.82	0.78	–	–	–
NDVI780	2015	–	–	–	–	–	–	0.79	0.71	0.70
	2016	–	–	–	–	–	–	0.79	0.64	0.69
NDVI800	–	0.69	0.60	0.57	0.88	0.82	0.84	–	–	–
	–	0.76	0.72	0.67	0.90	0.82	0.78	–	–	–
MTCI	2015	0.75	0.60	0.61	0.91	0.86	0.87	0.86	0.76	0.79
	2016	0.81	0.75	0.70	0.92	0.85	0.82	0.84	0.73	0.77
MCARI	2015	0.67	0.61	0.66	0.84	0.87	0.82	0.78	0.75	0.63
	2016	0.70	0.75	0.54	0.88	0.85	0.66	0.75	0.77	0.76
MSR	2015	0.72	0.56	0.57	0.90	0.83	0.85	0.83	0.73	0.75
	2016	0.73	0.73	0.67	0.90	0.82	0.78	0.81	0.68	0.74
DD/MSAVI	2015	0.70	0.52	0.46	0.82	0.67	0.73	0.80	0.70	0.73
	2016	0.78	0.69	0.70	0.92	0.79	0.77	0.74	0.56	0.60

*Hyperspectral vegetation indices (HVIs), Leaf dry weight (LDW), Leaf nitrogen concentration (LNC), Leaf nitrogen accumulation (LNA), Elongation (EL), Booting (BT), Heading (HD). Simple Ratio (SR), Normalized difference (ND), Normalized difference index (NDI), Normalized difference vegetation index (NDVI), The MERIS terrestrial chlorophyll index (MTCI), Modified chlorophyll absorption in reflectance index (MCARI), Modified simple ratio (MSR), Double difference index (DD), Modified soil adjusted vegetation index (MSAVI), DD/MSAVI.*

### Stepwise Multiple Linear Regression Analysis

Power regression was used to develop a static model between CN status indicators and HVIs as shown in the supplementary Table 1. SMLR was used to develop the dynamic models between CN status indicators and HVIs (Table 4).

**Table 4:** Stepwise multiple linear regression models for estimation of leaf nitrogen status indicators (LDW, LNC, and LNA) over growth stages of rice.

Stage	Y	Regression equation	*R*^2^_C_	*RMSEC*
Elongation	LDW	Y = 295.12 × SR_777/759_ + 104.851 × DD/MSAVI-2924.72	0.78	48.86
	LNC	Y = 0.15298 × SR_810/660_ – 2.0902	0.78	0.58
	LNA	Y = 0.616 × SR_810/660_ – 4.031 × MCARI-7.0435	0.72	2.63
Booting	LDW	Y = 4.496 × SR_810/660_ + 0.543 × LDWE∙ + 11.09 × SR_810/560_ – 5.596 × ND_860-560_ – 20.151	0.93	28.20
	LNC	Y = –0.332 × MCARI+0.596 × LNCE^∗^ + 26.516 × SR_768/750_ – 23.141 × DD/MSAVI-27.907	0.94	0.33
	LNA	1.0276 × LNAE† + 0.0495 × ND_860-560_ – 0.5751	0.99	0.56
Heading	LDW	Y = 260.954 × ND_860-720_ + 0.939 × LDWB∙ – 2738.59 × SR_777/759_ + 3.673 × SR_810/660_ + 2715.49	0.87	0.45
	LNC	Y = 0.221 × MCARI + 0.477 × LNCE^∗^ + 0.162 × SR_810/560_ – 6.842 × NDI_850_ + 0.512 × LNCB^∗^ 0.352 × ND_759-732_ + 3.337	0.98	0.21
	LNA	Y = 0.476 × LNAE† + 0.4197 × SR_810/560_ – 12.084 × NDVI_780_ + 0.510 × LNAB^†^ + 5.917	0.99	0.45

*Leaf dry weight at elongation and booting • (LDWE) and (LDWB), Leaf nitrogen concentration at elongation and booting, ^∗^ (LNCE), and (LNCB), Leaf nitrogen accumulation at elongation and booting^†^ (LNAE) and (LNAB).*

The simple ratio, SR_777/759_, and DD/MSAVI showed a good estimation for LDW at the EL stage. At BT, SR_810/660_, SR_810/560_, and ND_860-560_, along LDW of EL estimate LDW better. The indices for the red and NIR regions (ND_860-720_, SR_777/759_, and SR_810/660_) at BT along LDW of booting provides the best estimate of LDW at heading. The SMLR model indicated that 93% to 87% of variability in LDW (at BT and HD, respectively) could be explained using 2–3 band based HVIs. For EL and BT, the first sensitive band chosen was red, followed by NIR and red edge. For HD, or across growth stages, NIR was always the first band chosen, followed by the red edge bands.

Among all the HVIs, SR_810/660_ estimated LNC best at EL. At BT, LNC was best estimated with MCARI, SR_768/750_, and DD/MSAVI along the LNC of EL. At HD, MCARI, SR_810/560_, NDI_850_, and ND_759-732_ gave the best explanation of LNC estimation along the LNC of elongation and booting. Overall, it was determined that 98 to 94% of the dynamic LNC (at BT and HD, respectively) was explained by SR_810/560_, SR_768/750_, ND_759-732_, and MTCI based on the green, red edge, and NIR regions. LNA variability at EL (72%) was explained in the HVIs sensitive in the blue, red, and NIR bands (SR_810/660_, MCARI). Variability at BT and HD (99%) was explained in the green, red, red edge, and NIR-based bands: SR_810/660_, ND_860-560_, NDVI_780_, and MCARI along LNA of EL and BT. Among all the HVIs, ND_860-560_ in the green and NIR regions along LNA of EL best fit the SMLR model for LNA estimation at BT.

### Validation of Dynamic Models

**FIGURE 5:**
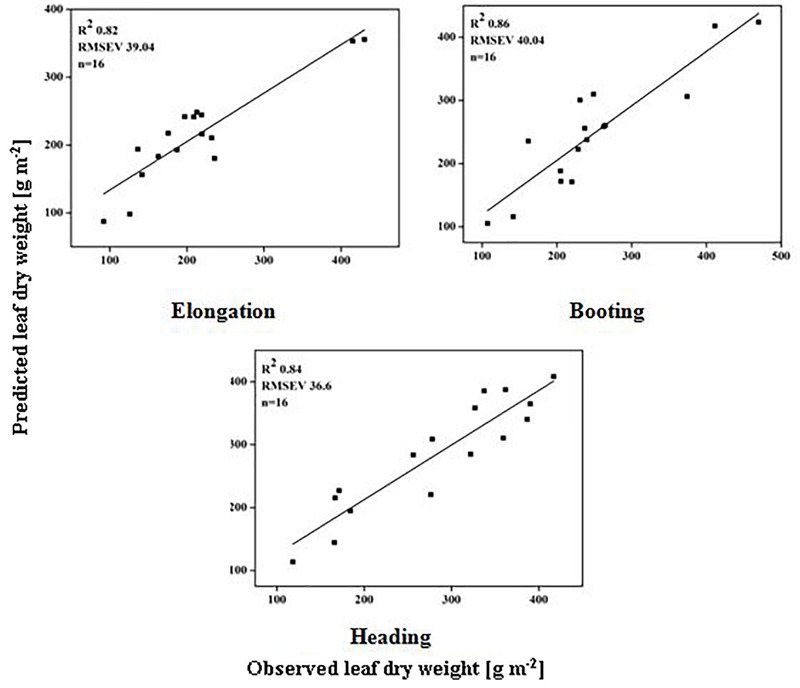
Relationship between predicted and observed LDW (g m^-2^) over phenological stages of rice.

**FIGURE 6:**
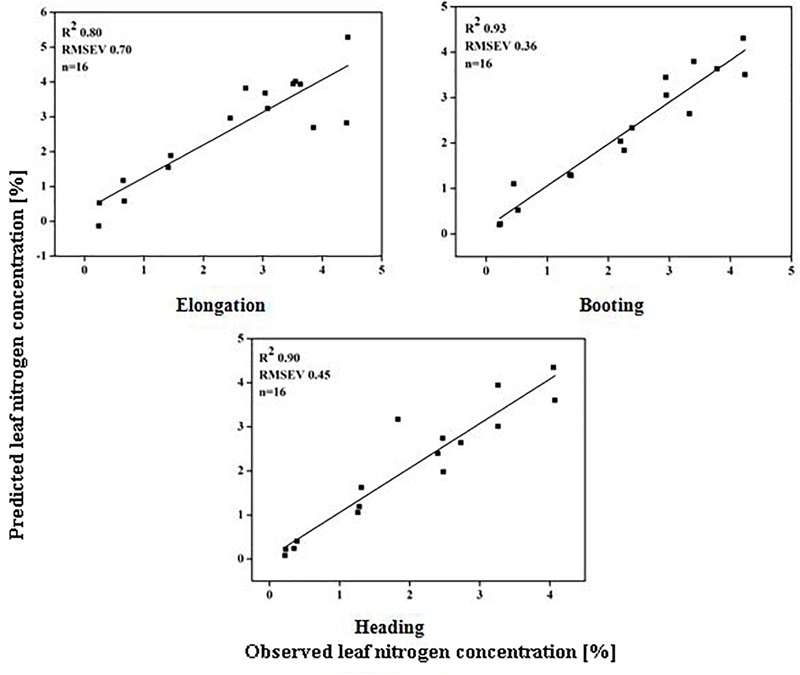
Relationship between predicted and observed leaf nitrogen concentration (%) over phenological stages of rice.

**FIGURE 7:**
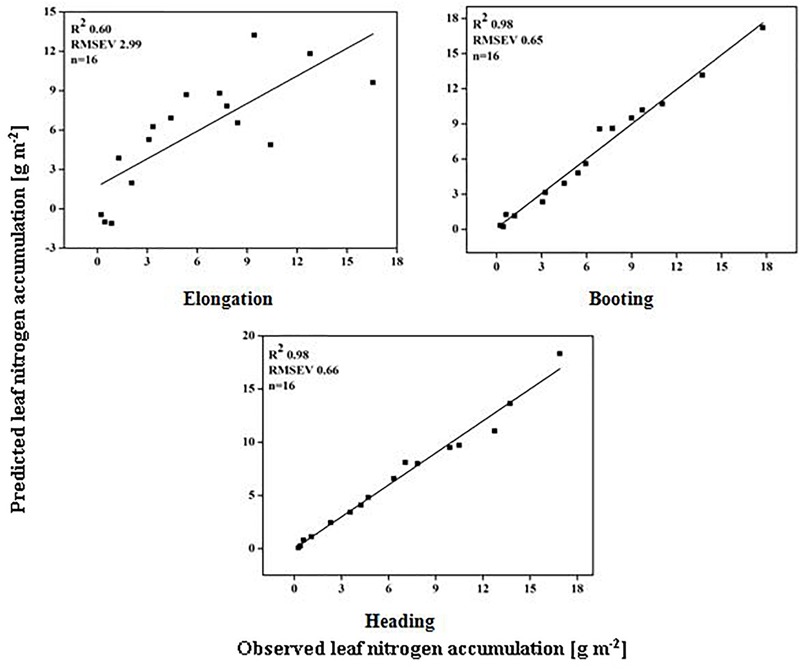
Relationship between predicted and observed leaf nitrogen accumulation (g m^-2^) over phenological stages of rice.

The models used above to describe the relationship between HVIs and CN status indicators were evaluated further using the validation data (Table 5). The SMLR models predicted LDW differently across three growth stages. The validation of the SMLR model for BT and HD performed better (*R*^2^ 0.86 and 0.84, respectively) than those for EL (*R*^2^ 0.82, *RMSEV* 39.04). At BT and HD, the SMLR models SR_810/660_, SR_810/560_, and ND_860-560_, and SR_777/759_, ND_860-720_, SR_810/660_ performed better than SR_777/759_ and DD/MSAVI did for estimating LDW. Our results showed that the LDW might be better estimated at all phenological stages, particularly BT, with a dynamic model using HVIs (Figure 5). The validation results indicated that the SMLR model and some HVIs (SR_810/560_, SR_768/750_, difference indices ND_759-732_, MCARI, and DD/MSAVI) could better predict LNC across all the growth stages. The SMLR model using HVIs (SR_768/750_, MCARI, and DD/MSAVI) along LNC of EL performed better (*R*^2^_val_ = 0.93 and *RMSEV* = 0.36) at BT. Therefore, the relationship between the estimated and observed LNC at BT was more prominent than at EL and HD (Figure 6). The validation results showed that at BT, the SMLR model and some HVIs (ND_860-560_, LNA of EL) were strongly correlated (*R*^2^_val_ = 0.98, *RMSEV* = 0.65) with prediction of LNA. For specific growth stages, the SMLR models performed better at BT and HD (*R*^2^_val_ = 0.98 and 0.98, *RMSEV* = 0.65 and 0.66, respectively) than at EL (*R*^2^_val_ = 0.60, *RMSEV* = 2.99) (Figure 7). The validation results confirm this observation.

**Table 5:** Validation of SMLR models for estimation of leaf nitrogen status indicators (LDW, LNC, and LNA) over growth stages of rice.

Stage	Y	Modeling set (*n = 32*)	*R*^2^_V_	*RMSE*V
Elongation	LDW	SR_777/759_, DD/MSAVI	0.82	39.04
	LNC	SR_810/660_	0.80	0.70
	LNA	SR_810/660_, MCARI	0.60	2.99
Booting	LDW	SR_810/660_, LDWE•, SR_810/560_, ND_860-560_	0.86	40.04
	LNC	MCARI, LNCE, SR7_68/750_, DD/MSAVI	0.93	0.36
	LNA	LNAE†, ND_860-560_	0.98	0.65
Heading	LDW	ND_860-720_, LDWB•, SR_777/759_, SR_810/660_	0.84	36.6
	LNC	MCARI, LNCE^∗^, SR_810/560_, NDI_850_, LNCB^∗^, ND_759-732_	0.90	0.45
	LNA	LNAE, LNAB^†^, SR_810/560_, NDVI_780_	0.98	0.66

*Leaf dry weight at elongation, booting, and heading • (LDWE), (LDWB), and (LDWH), Leaf nitrogen concentration at elongation, booting, and heading ^∗^ (LNCE), (LNCB), and (LNCH), Leaf nitrogen accumulation at elongation, booting, and heading ^†^ (LNAE), (LNAB), and (LNAH).*

## Discussion

In-season estimation of crop growth and N status are essential indicators for the precise management of N levels and grain yield (Ata-Ul-Karim et al., 2016, 2017b). The results of this study support the hypothesis that heterogeneous discrepancies in CSR over different phenological stages formulate several narrow band HVIs that have the potential to estimate crop growth and in-season N status.

Taking into account all the data from the two experiments in the present study, the significant effect of the N fertilizer on the CN status indicators (LDW, LNC, and LNA) has been widely reported (Chu et al., 2014; Yao et al., 2014; Ata-Ul-Karim et al., 2017a). The LDW increased when the rice studied in the present work was in the BT and HD stages (Figures 1A,B), in consensus with previous studies (Brandao et al., 2015; Guo et al., 2017). Our work revealed that LDW improved in EL, BT, and HD, but significant increases were observed at booting and heading in 2015 and 2016. The elevated N fertilization rate generally demonstrated higher LNC, yet a decline in the LNC was seen as LDW increased during the growth period (Figures 1C,D). The LNC was negatively affected by incremental N fertilization, with the advances in plant growth followed by tiller initiation to EL and MT. The decline in LNC during vegetative growth was attributable to the decrease in N concentration per unit leaf area for shaded leaves, in agreement with Wang et al. (2009). This is because the availability of LNC was influenced by the total N concentration in the paddy surface water and the N fertilization rate. Moreover, the proportion of self-shaded leaves with low N concentrations increased, and the proportion of the uppermost leaves with high N concentrations declined, optimizing canopy photosynthesis during canopy development corresponding to an optimization of N allocation in relation to light distribution (Ogawa et al., 2016). LNA is the product of LDW and LNC; therefore, plants with high LNC and low LDW at earlier growth stages may have similar LNA as those with low LNA and higher biomass at later growth stages. Thus, the growth stage is an important reference factor that must be taken into consideration when using LNA as an indicator for estimating crop N levels. LNA was significantly higher in rice that was more highly fertilized; this might be an effective way to regulate LNA, which was significantly affected by radiation absorption and CSR under different N rates in our study.

It was also revealed that LNA increased with increasing N fertilization along growth stages (Figures 1E,F). LNA was significantly higher in 2016 than the 2015 under same N application, because the field experiment had just begun in 2015. The actual soil fertility might compromise the dilution effect of concentrated plant N, ultimately affecting LNA during the crop growth stages. Wei et al. (2017) found similar responses, indicating that a higher LNA trend might be due to increased precipitation and radiation levels, and that this increased the dry matter and leaf area index, thereby increasing relative LNA levels. When this occurs during the crucial periods for leaf N uptake, from initiation of TL to end of HD, there is an improvement in crop yield and N accumulation from stem elongation. The increased N uptake at higher N fertilization levels might be a result of increased root growth and the subsequent absorption of more N from the soil, leading to the higher N accumulation reported by Fan et al. (2010). The application of more N at the seedling stage did not result in a higher N uptake. Zhang et al. (2007) found that the critical period for N uptake was from the initiation of tillering to the end of the heading stage, and similar results were observed in the current study. The effective regulation of N accumulation at booting to heading stages improved LNA at higher N fertilization rates that influence primarily the canopy spectral absorption and reflectance.

Variation in CSR during different phenological stages might be due to differences in N content. The rapid growth from the EL to the BT stage boosts N absorption, which leads to a higher reflectance relative to the high level of leaf N in the grain from the HD to the MA stages and N-remobilization from leaves to grain, which leads to leaf senescence. Strong absorption in the red region was relative to leaf chlorophyll levels, and reflectance in this region is also closely associated with leaf N status, and high reflectance in the NIR region is associated with the mesophyll structure in growing plants. Our previous work (Din et al., 2017) showed that at the vegetative stages (EL and BT) of rice, the canopy reflectance in the infrared (>760 nm) and visible (524–534, 583, 687, and 707 nm) regions were very prominent for leaf characterization. Variations (e.g., green-yellow) in leaf color and N remobilization might be the reason for incremental changes in reflectance in the short-wave NIR at the BT stage under different N rates. The character of the leaves and the canopy was significantly related to biomass, about which the spectral reflectance provides important information. Everard et al. (2012) noted that biomass estimation was more accurate in the 800–1680 nm spectral range than in the 450–950 nm range. The NIR band centered at 810–860 nm mainly carried information on LDW estimation.

In our study, the CSR sensitive spectral ranges 530–560, 680–700, and 700–780 nm were mainly reported for LDW estimation. The dense canopy led to a higher reflectance in the NIR regions, causing an increase in the green pigment, especially in leaves in the early stage. The CSR in the red edge (680–760 nm) regions proved to be effective for estimating chlorophyll content, total N, and total yield (Sharabian et al., 2014). The CSR, red edge, and NIR regions were found to be suitable to use to estimate LNC for different phenological stages, in agreement with previous research (Feng et al., 2014). Combining the CSR green band with red edge and NIR regions could result in a better index for correlating the LNC of rice, compared to the blue, red, and SWIR (Chen et al., 2010). Effective regulation of canopy N accumulation caused positive correlation of LNA in the NIR (720–900 nm) region. However, the red (670–790 nm) region predicted LNA better under varied N rates. Li et al. (2012) also found that red-edge indices were more sensitive for plant N estimation. LNA estimation sensitive bands were located in range of visible (550–750 nm) and NIR (750–860 nm) regions. Leaf irradiance correlated to photosynthesis would make it possible to investigate and estimate LNA in a non-destructive manner (Frak, 2002).

The dynamic pattern of LDW showed that the LDW increased linearly from the mid-vegetation to the mid-maturity period. As the biomass increased with growth stages, the number of HVIs were affected due to saturation that reduced the performance of univariate non-linear regression models constructed on these HVIs to estimate LDW. Several studies reported that SMLR using an increased number of wavebands could significantly improve the estimation of biophysical parameters compared to a single vegetation index (Dalponte et al., 2009; Miao et al., 2009; Yu et al., 2013). Using SMLR with HVIs provides increased flexibility and should achieve better results compared to HVIs using regression analysis, as in our study (Table 5). The use of canopy spectra for HVIs construction for N assessment depends mostly on the relationship between N and chlorophyll contents of the plant. Although the best performing HVIs increased the *R*^2^ for LNA estimation using univariate non-linear regression models, limitation still exist compared to SMLR models that produced the best estimates at the EL, BT, and HD stages. The SMLR model was capable of better predictions at heading by keeping the dynamic LNC and LNA over the phenological stages. The results agree with Huang et al. (2004), who reported that an SMLR provides reasonable information with which to extract leaf biochemistry information from leaf reflectance. Inconsistencies in the temporal pattern of rice canopy development showed again that phenology has a pronounced influence on the performance of HVIs used to estimate N concentrations, and have an especially larger influence in LNC and LNA (Bridhikitti and Overcamp, 2012).

At EL, the variation in biomass and canopy structure was less than that at BT and HD because the N in the top canopy leaves can be remobilized from shaded leaves at the bottom to the leaves above (Lemaire et al., 2008). Thus, greater variation in LNC and LNA was explained by the HVIs at the booting and heading stage. The influence of the N dilution effect made it difficult to support N related physiological parameters using only two band HVIs in univariate non-linear regression analysis. Therefore, SMLR models with a number of HVIs are required to provide a detailed illustration of LNC and LNA over phenology. The newly modified SR_768/750_ responded quite well to estimating dynamic LNC over phenology in SMLR models, in a manner similar to the method used by Campbell et al. (2007) study, who found that SR predominant for LNC estimations over the growth period. Our results confirmed findings by Grossman et al. (1996), who stated that SMLR could be successfully used for dynamic estimation of crop nutrient (N, P) status using hyperspectral reflectance. The NIR and red edge bands used to construct the HVIs, the NIR band sensitivity for leaf structure, and biomass were already reported. The single band analysis showed that the NIR bands had the best correlation with biomass and leaf N uptake, especially at heading and across growth period. Physiologically, canopy leaves respond to increased soil N supply by accumulating leaf N, which disturbed the chlorophyll pigment and photosynthetic enzymes to enhance the carbon assimilation in plant growth (Zhao et al., 2005b). Zhan et al. (2013) utilized SMLR to evaluate the relationship of phenanthrene uptake and root morphological and compositional characteristics in a manner similar to the methods we used in our study. The patterns of plant N uptake differ considerably between N fertilization over the EL, BT, and HD stages, and could successfully quantify the dynamic pattern for estimating CN status indicators over phenology using SMLR models.

## Conclusion

The CSR in the NIR (720–900 nm), visible (400–720 nm), and (560–710 nm) regions demonstrated the construction of functional HVIs that could characterize rice leaves and estimate LDW, LNC, and LNA primarily at three phenological stages. The current study formulates the new SR_777/759_, SR_768/750_ HVIs to predict LDW, LNC and LNA at the elongation, booting, and heading stages, based on these sensitive regions. The SMLR model efficiently estimates fluctuations in the LDW, LNC, and LNA over the latter two phenological stages under different N rates. It also showed a significantly better performance compared to univariate non-linear regression models, as the *R*^2^ and RMSE values were **0.93** and **28.20** for LDW; **0.98** and **0.21** for LNC; **0.99** and **0.45** for LNA estimates, respectively, over mid-vegetative and early reproductive stages in the life cycle of a rice plant.

Moreover, as LDW progressed from heading to maturity, there was a prominent negative decrease in LNC from elongation to maturity, and improvement in LNA, from tillering to booting under N fertilization rates, could be predicted efficiently using an SMLR model. The dynamic LDW during elongation and booting and the pattern of LDW over phenological stages are successfully quantified using HVIs when compared to univariate non-linear regression models that can only estimate LDW at a specific stage. The SMLR model more effectively predicted the dynamic LNC and LNA over the growth period across the phenological stages, including the heading stage, compared to the univariate non-linear regression model at each stage.

Our research shows that SMLR models have the potential to estimate the dynamic LNA, LNC, and LDW at different phenological stages using specific HVIs, when compared to univariate non-linear regression using a number of HVIs. The findings of this study could be used as a direct theoretical and practical reference for using hyperspectral remote sensing to conduct non-destructive monitoring and precise estimation of N-indices during production of rice.

## Author Contributions

MD and SW formulated the original idea designed the experiments, and wrote the manuscript. MD and JM performed the field experiments and hyperspectral measurements. MD and SH performed the laboratory analyses. MD and MT reviewed the manuscript. SA-U-K and MR provided editorial advice.

## Conflict of Interest Statement

The authors declare that the research was conducted in the absence of any commercial or financial relationships that could be construed as a potential conflict of interest.

## Abbreviations

AT: after transplanting
BT: booting
CMR: chlorophyll meter readings
CN: canopy nitrogen
CSR: canopy spectral reflectance
DD: double difference index
EL: elongation
FRTM: field-spectro-radiometer
HD: heading
HVIs: hyperspectral vegetation indices
LDW: leaf dry weight
LNA: leaf N accumulation
LNC: leaf N concentration
MCARI: modified chlorophyll absorption in reflectance index
MSR: modified simple ratio
MT: maturity
MTCI: The MERIS terrestrial chlorophyll index
N: nitrogen
ND: normalized difference
NDI: normalized difference index
NDVI: normalized difference vegetation index
NIR: near infrared
PNC: plant nitrogen content
PP: pre-planting
RMSE: root mean square error
RMSE_cal_: root mean square error of calibration
RMSE_val_: root mean square error of validation
RMSEV: root mean square error of validation
SMLR: stepwise multiple linear regression
SR: simple ratio index
SSP: single super phosphate
TL: tillering
