# Supplementary material for: Estimation of Dynamic Canopy Variables Using Hyperspectral Derived Vegetation Indices Under Varying N Rates at Diverse Phenological Stages of Rice
Source: Front Plant Sci. 2019 Jan 15;9:1883. doi: 10.3389/fpls.2018.01883 (PMC6340937; doi:10.3389/fpls.2018.01883)
Supplement: Supplementary file 1 [file Table_1.docx]

Supplementary Material

Estimation of dynamic canopy variables using hyperspectral derived vegetation indices under varying N rates at diverse phenological stages of rice

Mairaj Din^1, 2, 3^, Jin Ming^1, 2^, Sadeed Hussain^1, 2^, Syed Tahir Atta-Ul-Karim^4^, Muhammad Rashid^5^,Muhammad N. Tahir^6^, Zhihua Shi^1,2^ ShanqinWang^1, 2,*^

*** Correspondence:** Corresponding Author: sqwang@mail.hzau.edu.cn (S. Wang)

## Development of relationship for canopy nitrogen status indicators (LDW, LNC and LNA) over growth stages

Among all the possible HVIs, 20 were based on the single, double, and triple wave bands from 400–900 nm; these were used to develop the static relationship between CN status indicators (LDW, LNC, and LNA) at the three phenological stages (EL, BT, and HD).

Among the different simple univariate regression models, such as the linear, exponential, and power relationships developed for the HVIs and CN status indicators, only the univariate non-linear power model resulted in the maximum coefficient of determination (*R^2^*). Therefore, this relationship was used to compute the variation in the HVIs at three stages during 2015 and 2016 data provided in supplementary Table 1. The results showed that the top 15 HVIs performed well for CN status indicators across the year and for growth stages among 20 HVIs. Ten HVIs were specific for LDW (*R^2^*> 0*.65*) and eleven were closely related to LNC (*R^2^*> 0.90) and LNA (*R^2^*> 0.85). Moreover, the LNC and LNA related HVIs lie in sensitive regions ranging from 730–760 nm, 770–850 nm, 810–900 nm, 550–750 nm, and 750–860 nm during 2015 and 2016. The performance of HVIs also differed according to the growth stage.

The top 10 indices had stronger relationships with LDW at the EL (*R^2^* = 0.68–0.50), BT (*R^2^* = 0.60–0.35), and HD (*R^2^* = 0.50–0.17) stages, but none of the indices were the same. The results demonstrated that the SR_810/560_, NDI,NDI_850_, NDVI_800,_ MSR, and MTCI provide the best relationship to estimate LDW over three phenology stages (EL, BT and HD). For LNC, the SR_777/750_, SR_810/560_, NDI_850_, NDVI_800_, MTCI, and MSR showing the best estimation at EL, BT and HD stages of rice growth. Among the 11 specific HVIsthat correlated with LNA, SR_768/750_, SR_777/550_,ND_860/560,_ NDI_860-720,_ NDVI_780_, MTCI, and MSR performed the best at the three phenological stages in the present study. For LNA, SR_768/750_ (*R^2^*> 0.75) was consistently among the top indices for the three phenological stages data provided in **supplementary Table 1**.

|  |  | **LDW** | | | | | | **LNC** | | | | | | **LNA** | | | | | |
| --- | --- | --- | --- | --- | --- | --- | --- | --- | --- | --- | --- | --- | --- | --- | --- | --- | --- | --- | --- |
|  |  | **Elongation** | | **Booting** | | **Heading** | | **Elongation** | | **Booting** | | **Heading** | | **Elongation** | | **Booting** | | **Heading** | |
| **HVIS** |  | **Model Fitting** | ***R^2^*** | **Model Fitting** | ***R^2^*** | **Model Fitting** | ***R^2^*** | **Model Fitting** | ***R^2^*** | **Model Fitting** | ***R^2^*** | **Model Fitting** | ***R^2^*** | **Model Fitting** | ***R^2^*** | **Model Fitting** | ***R^2^*** | **Model Fitting** | ***R^2^*** |
| SR768/750 | 2015 |  | -- |  | -- |  | -- | 0.0415x^27.46^ | 0.84 | 0.0246x^30.12^ | 0.82 | 0.0073x^45.12^ | 0.79 | 0.020x^35.79^ | 0.86 | 0.015x^38.02^ | 0.80 | 0.004x^57.17^ | 0.75 |
|  | 2016 |  | -- |  | -- |  | -- | 0.0555x^33.62^ | 0.86 | 0.0249x^33.98^ | 0.84 | 0.0463x^29.30^ | 0.66 | 0.024x^46.56^ | 0.90 | 0.025x^42.95^ | 0.84 | 0.056x^37.75^ | 0.70 |
| SR 777/750 | 2015 |  | -- |  | -- |  | -- | -- | -- | -- | -- | -- | -- | 0.024x^31.63^ | 0.85 | 0.015x^38.02^ | 0.79 | 0.004x^49.53^ | 0.72 |
|  | 2016 |  | -- |  | -- |  | -- | -- | -- | -- | -- | -- | -- | 0.023x^42.03^ | 0.90 | 0.025x^38.22^ | 0.84 | 0.053x^33.91^ | 0.64 |
| SR777/759 | 2015 | 68.155x^17.87^ | 0.60 | 85.343x^13.59^ | 0.41 | 73.115x^23.34^ | 0.38 | -- | -- | -- | -- | -- | -- | -- | -- | -- | -- | -- | -- |
|  | 2016 | 0.8739x^0.03^ | 0.68 | 0.8804x^0.03^ | 0.47 | 0.939x^0.02^ | 0.17 | -- | -- | -- | -- | -- | -- | -- | -- | -- | -- | -- | -- |
| SR810/560 | 2015 | 18.093x^0.97^ | 0.60 | 28.871x^0.86^ | 0.38 | 24.757x^1.13^ | 0.34 | 0.0011x^3.31^ | 0.85 | 0.0008x^3.48^ | 0.67 | 0.0002x^4.65^ | 0.72 | 0.000x^4.17^ | 0.84 | 0.000x^4.20^ | 0.65 | 5E-05x^5.60^ | 0.65 |
|  | 2016 | 0.1584x^0.68^ | 0.68 | 0.3815x^0.60^ | 0.59 | 0.8792x^0.42^ | 0.41 | 0.0013x^3.41^ | 0.87 | 0.0001x^3.94^ | 0.80 | 0.0008x^3.26^ | 0.58 | 3E-05x^5.07^ | 0.86 | 3E-05x^4.82^ | 0.78 | 0.000x^4.06^ | 0.58 |
| SR810/660 | 2015 | 14.62x^0.70^ | 0.51 | 28.19x^0.57^ | 0.32 | 24.093x^0.76^ | 0.30 | 0.0003x^2.58^ | 0.85 | 0.0005x^2.40^ | 0.64 | 0.0001x^3.26^ | 0.66 | 4E-05x^3.27^ | 0.83 | 0.000x^2.97^ | 0.59 | 3E-05x^4.02^ | 0.61 |
|  | 2016 | 0.2752x^0.81^ | 0.62 | 1.8749x^0.56^ | 0.36 | 1.6974x^0.53^ | 0.30 | 0.0001x^2.89^ | 0.92 | 4E-05x^2.76^ | 0.56 | 0.0017x^1.85^ | 0.42 | 9E-06x^3.64^ | 0.67 | 1E-05x^3.41^ | 0.53 | 0.000x^2.42^ | 0.42 |
| ND860-560 | 2015 |  | -- |  | -- |  | -- | -- | -- | -- | -- | -- | -- | 0.000x^3.37^ | 0.85 | 0.000x^3.21^ | 0.63 | 5E-05x^5.60^ | 0.65 |
|  | 2016 |  | -- |  | -- |  | -- | -- | -- | -- | -- | -- | -- | 3E-05x^5.07^ | 0.86 | 0.000x^3.15^ | 0.66 | 8E-05x^5.54^ | 0.68 |
| ND860-720 | 2015 |  | -- |  | -- |  | -- | -- | -- | -- | -- | -- | -- | 220.4x^3.915^ | 0.71 | 284.3x^4.16^ | 0.72 | 7374.x^5.69^ | 0.70 |
|  | 2016 |  | -- |  | -- |  | -- | -- | -- | -- | -- | -- | -- | 4254.x^5.63^ | 0.83 | 9161x^6.48^ | 0.82 | 279.0x^3.48^ | 0.60 |
| ND759-732 | 2015 |  | -- |  | -- |  | -- | 177.85x^2.91^ | 0.75 | 215.04x^3.14^ | 0.76 | 2495.8x^4.00^ | 0.72 | -- | -- | -- | -- | -- | -- |
|  | 2016 |  | -- |  | -- |  | -- | 0.0203x^9.48^ | 0.89 | .0066x^10.39^ | 0.85 | 0.0145x^9.09^ | 0.72 | -- | -- | -- | -- | -- | -- |
| NDI850 | 2015 | 341.34x^4.14^ | 0.57 | 444.76x^2.74^ | 0.48 | 651.42x^2.76^ | 0.38 | 27.583x^14.68^ | 0.88 | 36.064x^10.20^ | 0.75 | 113.27x^11.15^ | 0.75 | -- | -- | -- | -- | -- | -- |
|  | 2016 | 65.56x^2.34^ | 0.62 | 758.39x^3.05^ | 0.57 | 880.66x^3.076^ | 0.48 | 40.699x^8.77^ | 0.91 | 65.688x^12.41^ | 0.81 | 38.327x^10.19^ | 0.67 | -- | -- | -- | -- | -- | -- |
| NDVI780 | 2015 |  | -- |  | -- |  | -- | -- | -- | -- | -- | -- | -- | 148.2x^17.30^ | 0.88 | 153.3x^16.63^ | 0.66 | 855.5x^18.66^ | 0.67 |
|  | 2016 |  | -- |  | -- |  | -- | -- | -- | -- | -- | -- | -- | 221.84x^15.06^ | 0.87 | 377.15x^23.41^ | 0.77 | 292.15x^19.64^ | 0.62 |
| NDVI800 | 2015 | 324.02x^4.80^ | 0.56 | 447.06x^2.81^ | 0.46 | 663.63x^2.79^ | 0.38 | 23.666x^17.23^ | 0.88 | 38.151x^10.59^ | 0.74 | 123.06x^11.26^ | 0.76 | -- | -- | -- | -- | -- | -- |
|  | 2016 | 665.56x^2.34^ | 0.62 | 780.68x^3.20^ | 0.58 | 911.42x^3.12^ | 0.49 | 40.699x^8.76^ | 0.91 | 71.309x^12.90^ | 0.82 | 40.36x^10.15^ | 0.67 | -- | -- | -- | -- | -- | -- |
| MTCI | 2015 | 111.21x^0.64^ | 0.50 | 135.91x^0.66^ | 0.49 | 213.96x^0.91^ | 0.52 | 0.0319x^3.61^ | 0.85 | 0.0207x^4.01^ | 0.78 | 0.0229x^4.95^ | 0.79 | 0.014x^4.703^ | 0.87 | 0.012x^5.068^ | 0.75 | 0.017x^6.225^ | 0.74 |
|  | 2016 | 116.73x^0.96^ | 0.66 | 95.463x^1.12^ | 0.62 | 107.34x^1.16^ | 0.51 | 0.0659x^3.58^ | 0.91 | 0.0163x^4.43^ | 0.84 | 0.0353x^3.85^ | 0.72 | 0.0769x^4.57^ | 0.90 | 0.0156x^5.54^ | 0.82 | 0.0379x^5.01^ | 0.73 |
| MCARI | 2015 | 111.21x^0.64^ | 0.50 | 135.91x^0.66^ | 0.49 | 213.96x^0.91^ | 0.52 | 0.4954x^2.34^ | 0.82 | 0.4298x^2.52^ | 0.78 | 1.2906x^3.15^ | 0.76 | 0.551x^2.974^ | 0.81 | 0.584x^3.170^ | 0.75 | 2.761x^4.058^ | 0.74 |
|  | 2016 | 823.04x^2.02^ | 0.54 | 190.78x^0.90^ | 0.59 | 253.6x^0.63^ | 0.31 | 113.45x^8.01^ | 0.89 | 0.238x^3.64^ | 0.84 | 0.5651x^2.31^ | 0.55 | 933.76x^10.03^ | 0.85 | 0.454x^4.54^ | 0.82 | 1.433x^2.94^ | 0.53 |
| MSR | 2015 | 63.458x^1.43^ | 0.59 | 87.046x^1.31^ | 0.43 | 122.47x^1.50^ | 0.36 | 0.0779x^4.91^ | 0.86 | 0.073x^5.10^ | 0.73 | 0.1338x^6.06^ | 0.75 | 0.049x^6.336^ | 0.87 | 0.063x^6.412^ | 0.69 | 0.163x^7.555^ | 0.69 |
|  | 2016 | 230.8x^0.63^ | 0.55 | 112.67x^1.57^ | 0.58 | 139.67x^1.54^ | 0.48 | 0.7411x^2.45^ | 0.89 | 0.0301x^6.28^ | 0.80 | 0.09x^5.014^ | 0.65 | 1.7105x^3.08^ | 0.86 | 0.0339x^7.86^ | 0.78 | 0.1256x^6.55^ | 0.66 |
| DD/MSAVI | 2015 | 1650.9x^0.78^ | 0.59 | 1275.7x^0.62^ | 0.39 | 629.23x^0.33^ | 0.29 | 2406.3x^2.41^ | 0.68 | 1083x^2.14^ | 0.52 | 65.932x^1.20^ | 0.48 | 39725x^3.191^ | 0.72 | 13816x^2.761^ | 0.52 | 414.8x^1.527^ | 0.46 |
|  | 2016 | 149.78x^1.29^ | 0.64 | 837.32x^0.32^ | 0.39 | 1401.6x^0.44^ | 0.61 | 0.1544x^4.82^ | 0.92 | 124.39x^1.37^ | 0.62 | 82.767x^1.23^ | 0.61 | 0.2313x^6.11^ | 0.90 | 1041.5x^1.69^ | 0.59 | 1160.1x^1.67^ | 0.67 |

**Table 1 Coefficient of determination (R^2^) of static models between HVIs and leaf nitrogen status indicators (LDW, LNC and LNA) over growth stages of rice in 2015 and 2016**
